# Factors Associated With Patient Satisfaction After Nasal Breathing Surgery

**DOI:** 10.1001/jamanetworkopen.2020.1409

**Published:** 2020-03-23

**Authors:** Ashley M. Nassiri, Scott J. Stephan, Liping Du, William R. Ries, Roland D. Eavey

**Affiliations:** 1 Department of Otolaryngology, Vanderbilt University Medical Center, Nashville, Tennessee; 2 Department of Biostatistics, Vanderbilt University Medical Center, Nashville, Tennessee

## Abstract

This cohort study examines subjective patient-reported outcomes after nasal breathing surgery and identifies factors associated with patients’ perception of the procedure’s value.

## Introduction

Although surgical interventions are intended to improve patients’ symptoms, such interventions may not consistently achieve this goal or be cost-effective.^1^ Patient-reported outcomes become increasingly important in disease processes relying on subjective symptoms before and after intervention.^2,3^ Nasal obstruction surgery, a commonly performed procedure, is a fitting setting to test the efficacy of patient-reported outcomes for elective surgery with subjective outcome measurements. The validated Nasal Obstruction Symptom Evaluation (NOSE) instrument is 1 example of a patient-reported outcome that evaluates the severity of nasal obstruction from the patient perspective.^4^ The aims of this cohort study were to (1) report subjective patient-reported outcomes after nasal breathing surgery and (2) identify variables associated with patients’ perception of value.

## Methods

After approval by the institutional review board of Vanderbilt University, the cohort of patients who underwent nasal breathing surgery (septorhinoplasty) from 2014 to 2018 and provided oral informed consent were prospectively surveyed both preoperatively and postoperatively. Surveys were filled out on an electronic tablet and consisted of the NOSE instrument, demographic questions, and patient satisfaction questions. The NOSE score was scaled to 100, with a score of 0 reflecting no symptoms of nasal obstruction. There is no validated survey that measures patient satisfaction after nasal breathing surgery; thus, we developed questions (such as “based on your nasal breathing now, was this surgery worth it?”) to broadly capture patient satisfaction with the global experience, including their assessment of outcome and cost. The threshold for statistical significance was set at 2-tailed *P* < .05. A locally weighted scatterplot smoothing regression line was generated for postoperative NOSE or “worth it” score against survey time to demonstrate the trend with time. All analyses and graphs were generated using R version 3.5.0 (R Project for Statistical Computing).

## Results

**Figure 1.**
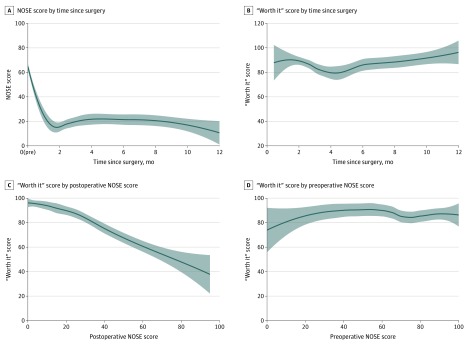
Changes in Nasal Obstruction Symptom Evaluation (NOSE) and Patient Satisfaction Scores Over Time A, The NOSE score decreased significantly postoperatively, indicating decreased symptom severity after surgery. B, When asked questions such as “based on your nasal breathing now, was this surgery worth it?” to determine patient satisfaction, patients rated a mean satisfaction score of 87.4% postoperatively, which was durable through 1 year after surgery. C and D, The satisfaction score was associated with the postoperative NOSE score (C) but not the preoperative NOSE score (D). The line indicates the locally weighted scatterplot smoothing regression line; shaded areas, 95% confidence intervals.

**Figure 2.**
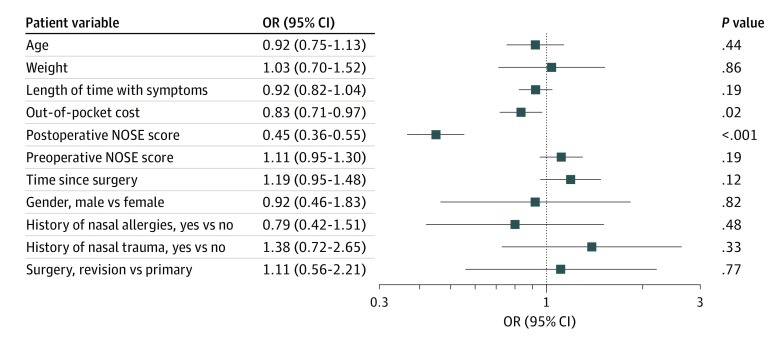
Patient Variables Associated With Patient Satisfaction Scores The postoperative Nasal Obstruction Symptom Evaluation (NOSE) score and out-of-pocket cost were statistically significantly associated with patient satisfaction. OR indicates odds ratio.

A total of 256 patients (55.1% female; median [interquartile range] age, 38.5 [29.0-52.2] years) underwent surgery for nasal obstruction, completing both preoperative and postoperative surveys. The median (interquartile range) NOSE score improved from 70 (50-80) preoperatively to 15 (5-30) postoperatively (*P* < .001) and was durable over 1 year (Figure 1). The mean (SD) patient satisfaction (“worth it”) score was 87.4% (20.2%). By univariate analysis, a higher postoperative NOSE score, indicating worse nasal obstructive symptoms, was significantly associated with decreased patient satisfaction (Spearman coefficient ρ = −0.54; *P* < .001) (Figure 1C). However, patient satisfaction was not associated with the preoperative NOSE score (Spearman coefficient ρ = −0.11; *P* = .12) (Figure 1D). A greater patient out-of-pocket cost was significantly associated with a lower patient satisfaction score (odds ratio, 0.83; 95% CI, 0.71-0.97; *P* = .02). There was no evidence of association between patient satisfaction and other patient demographic factors (Figure 2). There was no statistically significant interaction between out-of-pocket cost and postoperative NOSE score.

## Discussion

Through the use of patient-reported outcomes, this study identified patient characteristics that were associated with patient satisfaction following nasal breathing surgery. Specifically, a lower postoperative NOSE score, reflecting decreased symptoms after surgery, was positively associated with patient satisfaction, while a higher patient out-of-pocket cost was found to be negatively associated with patient satisfaction. Although out-of-pocket cost does not directly affect nasal obstruction symptoms, financial aspects have been shown to be a reliable method of determining the impact of preoperative morbidities on patients’ quality of life.^5^ Since out-of-pocket cost can be determined preoperatively, this factor may inform patient selection and preoperative counseling to maximize value for the patient.

This study has limitations that warrant discussion. The patient satisfaction measures used in this survey were purposefully broad-based questions designed to capture global measures of satisfaction; there was no direct assessment of individual aspects that may affect patient satisfaction, such as service delivery and recovery time. Additionally, future studies will evaluate NOSE scores of a control group for comparison.

Given that nasal obstruction is a subjective patient concern with subjective symptom measurements, the evaluation of surgical outcomes from the patient perspective is critical. Importantly, the surgeon’s opinion of surgical outcomes and patient satisfaction after nasal surgery do not necessarily align.^6^ Patient-reported outcomes, such as the NOSE score and patient satisfaction questions, offer clinicians instruments by which to capture surgical outcomes and patient-perceived value. Physicians who emphasize patient-centered outcomes contribute to a more efficient and meaningful model for health care delivery.
